# Speech and language therapist‐led clinics for low‐risk suspected head and neck cancer referrals: A qualitative study of ear, nose and throat surgeons’ views

**DOI:** 10.1111/1460-6984.13137

**Published:** 2024-12-11

**Authors:** Louise C. Occomore‐Kent, John C. Hardman, Justin W. G. Roe, Paula Bradley, Paul N. Carding, Joanne M. Patterson

**Affiliations:** ^1^ City St George's, University of London London UK; ^2^ The Royal Marsden Hospital NHS Foundation Trust London UK; ^3^ The Royal Marsden Hospital NHS Foundation Trust Imperial College Healthcare NHS Trust and Imperial College London London UK; ^4^ University of Sunderland Sunderland UK; ^5^ Oxford Institute of Nursing Midwifery and Allied Health Research Oxford UK; ^6^ University of Liverpool & Liverpool Head and Neck Centre Liverpool UK

**Keywords:** assessment, dysphagia, head and neck cancer, interview, oncology, otolaryngology, speech therapy, voice disorders, voice

## Abstract

**Background:**

Over 200,000 patients are referred onto the suspected head and neck cancer (HNC) pathway annually in the UK, with around 3% receiving a cancer diagnosis. With new HNC advancements in identifying patients at low risk of a cancer diagnosis, one proposal is a speech and language therapy (SLT)‐led first point of contact clinic for low‐risk patients presenting with voice or swallowing symptoms.

**Aims:**

To explore ear, nose and throat (ENT) surgeons’ views regarding this model.

**Materials & Methods:**

The study used a qualitative study design using semi‐structured interviews. Transcribed responses were analysed using thematic analysis.

**Outcomes & Results:**

A total of 11 UK ENT surgeons participated. Themes included the positives and challenges of the proposed model; existing facilitators that increase the likelihood of success; and the requirements for successful implementation. Service variation impacted level of interest. Waiting times were problematic at some sites more than others; SLT workforce issues were of differing prevalence; SLT competence and interest differed; and support from organizational leaders varied. Participants’ personal views also varied, for example, acceptability of the model to patients, or what governance structure is required. There was no single SLT‐led clinic model suitable for all centres; however, some general principles were identified to inform further evaluation and implementation of this model.

**Conclusions & Implications:**

Principles included the deployment of SLTs experienced in laryngeal examination and flexible nasendoscopy; dedicated job plans and workforce; professional and regulatory body recognition and support; and clear training, supervision structure and job description for the role. Service outcomes, training protocol and competencies require robust evaluation.

**WHAT THIS PAPER ADDS:**

**What is already known on this subject**

- During the SARS‐CoV‐2 pandemic, a risk calculator tool was developed to identify patients at highest risk of cancer who were referred to the 2‐week‐wait (2ww) ENT pathway. These patients require urgent face to face ENT assessment. Lower risk patients with voice and swallowing difficulties still require management of their symptoms and an SLT‐led clinic model is one potential solution for this population. While SLTs’ views of this model have been explored with a UK‐wide survey and focus groups, ENT surgeons’ perspectives are yet to be investigated.

**What this study adds to the existing knowledge**

- This study explores ENT surgeons’ views regarding SLT‐led clinics for patients who are referred through the 2ww ENT pathway, stratified as being at low risk of cancer, and with voice and/or swallowing symptoms of likely benign aetiology. Participants shared many positive perceptions of the clinic along with factors that they felt would enhance its success (facilitators). Key requirements for the success of this clinic model were also highlighted, along with challenges that need to be addressed.

**What are the practical and clinical implications of this work?**

- The need for this clinic model varied by centre. However, general principles included the importance of a dedicated workforce with pre‐existing relevant expertise; and a clear role profile, training process and competencies framework. Professional, statutory and regulatory bodies’ recognition of the role is required, and robust evaluation of the model is necessary.

## INTRODUCTION

Over 275,000 patients are referred onto the suspected head and neck cancer (HNC) pathway annually in the UK, with less than 3% of referrals leading to a diagnosis of cancer (National Disease Registration Service (NDRS, 2024). Up to a third of patients are referred with hoarseness as their primary symptom (Tikka et al., 2016). While persistent hoarseness may indicate a cancer diagnosis, it is also common in many benign conditions for which speech and language therapists (SLTs) are often the first line for treatment. The Faster Diagnosis Standard's Head and Neck pathway guidance (NHS, 2024), requiring cancer to be diagnosed or ruled out within 28 days of referral, has anecdotally placed significant pressure on secondary care services, largely delivered by ear, nose and throat (ENT) specialists, alongside maxillofacial surgeons, where appropriate. Coinciding with the COVID‐19 pandemic, which also placed huge pressures on ENT teams, these factors have driven novel models of service delivery for high‐risk patients with suspected HNC (Metcalfe et al., 2022; Warner et al., 2020). Consequently, there are an ever accumulating number of low‐risk patients on the suspected HNC pathway who require specialist assessment and care. People diagnosed with HNC experience one of the longest waiting times for treatment compared with other primary cancer sites in England as of July 2024 (National Health Service England (NHSE), 2024).

Many patients with benign conditions would benefit from SLT management of the symptoms that prompted their referral. However, they often experience a long and convoluted pathway before reaching these therapists. To address this delay, as well as improve capacity within secondary care, some UK centres are exploring alternative methods of service delivery by triaging patients referred on the suspected HNC pathway, who are felt to be at a low risk of cancer, and who have symptoms of dysphonia and/or dysphagia, directly to SLT‐led clinics. First‐point‐of‐contact‐SLT‐led clinics have already been shown to dramatically reduce waiting lists in Australia (Schwarz et al., 2021), with up to 70% of patients discharged from the pathway by SLTs without requiring ENT and with a consequential reduction in service cost (Payten et al., 2020). ‘Skill mix approaches’ are a key recommendation in a report by Cancer Research UK (2024) to address current diagnostic service challenges in England, further supporting this approach. Exploration of nurse‐led clinics is also emerging (Byrne et al., 2024) in response to the HNC pathway challenges. Some positive outcomes have been outlined including no missed diagnoses when nurses performed recorded nasendoscopy assessments for remote assessment by ENT surgeons (Metcalfe et al., 2024).

The consequences of the COVID‐19 pandemic led to the widespread adoption of a novel risk calculator (Paleri et al., 2020) to aid stratification of suspected HNC referrals into low‐ and high‐risk categories. This aimed to reduce unnecessary face‐to‐face, aerosol‐generating examinations for lower risk patients but has also provided validated support for adoption of remote triage into clinical pathways (Hardman et al., 2021). Low‐risk patients, in the context of the current study, are defined as patients who are stratified as being at low risk of cancer based on such a risk stratification tool. Effective triage of low‐risk patients, alongside increased service pressures, has presented a unique opportunity for SLTs to extend their role in ENT.

The views of SLTs in the UK have been investigated (Bradley & Patterson, 2021; Occomore‐Kent et al., 2021) and have shown high potential for the role from the SLT perspective. SLTs expressed a high level of interest in the extension of their role in this area. SLTs raised some issues they would wish to see addressed if this service becomes commonplace, including: professional and regulatory body recognition; remuneration to reflect increased responsibility; and clear training and supervision structure. Despite SLT‐led clinics emerging in a number of UK centres. published outcomes are limited. Two centres however report positive patient satisfaction and clinical outcomes with no adverse events (Butler et al., 2023; Occomore‐Kent & Slade, 2021). A survey of SLTs showed support for delivering low‐risk SLT‐led clinics but highlighted the importance of training and supervision (Bradley & Patterson, 2021) for successful implementation. More widespread adoption of SLT‐led clinics is only possible with the full support of ENT surgical colleagues.

### Objective

This study explored ENT surgeons’ views on the development and deployment of SLT‐led low‐risk suspected HNC clinics in the UK.

## MATERIALS AND METHODS

This was a qualitative study design, using semi‐structured interviews. Ethical approval was granted by the author's higher education institution.

### Sampling

Participants were included if they were consultant or specialty registrar ENT surgeons and currently involved in the clinical pathway of patients referred with suspected HNC. They were approached via professional organizations including the British Association of Head and Neck Oncologists (BAHNO) and the British Voice Association (BVA). This was followed by a snowballing approach whereby participants recruited additional research participants. Purposive sampling ensured suitability and diversity among participants, to capture varying models of extended role clinics such as new and well‐established clinics and clinics using a variety of non‐medical professionals.

Sample size was determined by information power (Malterud et al., 2016) where the research team conceded that there were no new themes identified from the data after three consecutive interviews.

### Procedure

In‐depth semi‐structured interviews took place between 13 July and 21 December 2021, each lasting up to 72 min. Interviews were based on flexible topic guides derived from the literature, informed by normalization process theory (NPT) framework (Murray et al., 2010) and expert opinion. Interviews were conducted by videoconferencing by an ENT surgeon or a general practitioner (GP) with a specialist interest in ENT. Interviews were recorded, transcribed, and anonymised. Reflexive thematic analysis, as described by Braun & Clarke (2013) was used for data analysis. Data were repeatedly read and coded by a member of the research team. Analysis was iterative, with ongoing review by the research team to identify issues for further exploration, and development of codes throughout data collection. Findings were reported with reference to COREQ guidelines (Tong et al., 2007).

### Credibility of data and analysis

Interviews were conducted by an ENT surgeon or a GP with a special interest in ENT to promote candour from interviewees when sharing their views about the SLT profession in this role. The specific interviewer for each participant was purposefully selected to minimize prior direct working relationships with participants, to further ensure data integrity. All data were reviewed by the wider research team, including SLTs, a GP and an ENT surgeon, when coding and interpreting the data. Coding, analysis and interpretation were agreed by consensus. Analysis was completed once the data had been transcribed and de‐identified to further minimize bias.

## RESULTS

A total of 11 consultant ENT surgeons from the UK participated, with no refusals or withdrawals (Table 1). The geographical distribution of participants is provided in Table 2.

**TABLE 1:** Demographic data.

**Interview number**	**1**	**2**	**3**	**4**	**5**	**6**	**7**	**8**	**9** ^a^	**10**	**11**
Sub‐specialty	HN	HN	HN	HN	HN	HN	HN	Laryngology	Laryngology	HN	HN
Years as a consultant	10	8	2	22	29	21	19	25	5	5	2
Sex	Male	Male	Male	Male	Male	Male	Male	Male	Male	Female	Female
Approximate number of suspected HNC referrals per annum	2000	1560	2600	2750	3200	3600	Unknown	Unknown	1800	900	Unknown
Unit experience of non‐consultant‐led clinics	Nurse‐led clinics	Nurse‐led clinics	Nil	Nil	Nil	Nil	SLT‐led clinics	SLT‐led clinics	n.a.	Nil	Nil

*Note*:

^a^ All interviews were conducted by J.C.H., except interview 9 by P.B. HN, head and neck.

**TABLE 2:** Geographical data.

**UK region**	**Number of participants**
West Midlands	3
South East	3
North West	1
South West	1
North East	1
Northern Ireland	1
East Midlands	1

Four themes were developed from the data: *positives* (Table 3) and *challenges* (Table 5) of SLTs in this role, *facilitators* that support the model (Table 4), and *requirements* for successful implementation (Table 6). Responses within those themes were categorized into 15 sub‐themes. Direct quotations illustrate each subtheme and participant numbers for each quotation are given in brackets [P*x*]. Further example quotations are available on request from the corresponding author.

**TABLE 3:** Theme 1: Positives.

**Sub‐theme**	**Examples**
Governance	‘our speech therapists … have picked up two cancers. And they haven't missed anything, to my knowledge. If there's any doubt, they will ask’ [P8]
Training and supervision	‘speech and language colleagues who have expertise in FEES,^a^ and have a knowledge base … that they can build upon … crucial … that we don't forget them …’ [P9]
SLT competence	‘[SLTs] extremely meticulous in their assessment of the images. And they pick up things … that … even consultants sometimes don't always recognise’ [P8]
SLT interest	‘I would imagine, across the board, speech therapists would be pretty keen to develop this role. … When you've got good people, they always want to do more’ [P4]
Patient experience	‘the worst person really … to see these low‐risk patients, … is head and neck doctors. … Because ultimately you go, yes, it's not cancer … you've not … given them a specific diagnosis, which … is a poor outcome and poor patient care’ [P10]
MDT support	‘it's been hugely cooperative. … We've got a very good close working relationship with the Speech and Language therapists involved’ [P7]
Organizational support	‘we don't need to accrue any more consultants and 70 odd grand a year, we can just employ speech and language therapists. So, I think the trust will love it. And I can see them buying into this very quickly’ [P9]
Time and workload	‘we could be waiting for eight weeks to try and see a cord check. Whereas our SLT will see them at her next clinic. So it's been a hugely effective resource’ [P7]
Cost	‘the initial work we got back … said it was highly cost‐effective compared to running a consultant clinic’ [P7]
Efficiency	‘only 2% to 3% come back into ENT’ [P7]
Equipment and resources	‘They've got equipment and they use stroboscopy’ [P8]
Workforce and sustainability	‘we've always had very active speech therapy departments, relatively well funded’ [P4]
Service models	‘it's very easy to pick up the ones … going to have definitive glottis cancer … in time I can see, even the speech and language clinicians … taking that initial history … referring the more concerning ones directly back. I can see that reversing in time’ [P9]
Precedent	‘we already do this … started a parallel Speech and Language service now about two years ago. … That worked very successfully’ [P7]
Need	‘what you're proposing I think is great … our benign voice pathology patients probably don't get the best deal at our end’ [P2]

*Note*: ^a^Flexible endoscopic evaluation of swallowing (FEES).

**TABLE 4:** Theme 2: Facilitators.

**Sub‐theme**	**Examples**
Governance	‘if you're going to have globus patients, then there are certain indicators you can use to pick up the low‐risk ones’ [P4]
Training and supervision	‘We set up a preparation for about a year beforehand where we trained a Speech and Language therapist’ [P7]
SLT competence	‘They've seen it before, they can recognise the patterns. They've got equipment … they use stroboscopy … so they have a much better understanding and have a different mindset, not all lesions on the vocal cords need a biopsy’ [P8]
SLT interest	‘SLTs have come forward in all those departments and said, look, we'd love to do this, this could be really interesting’ [P7]
Patient experience	[regarding a Physicians Associate in the role] ‘they've been very happy … she's very competent. Very personable. … We've not had any complaints or negative feedback’ [P3]
MDT support	‘it's, obviously, been done in discussion with all our head and neck consultants and they're very happy’ [P8]
Organizational support	‘We're working towards … tariffs … linked to the procedure. … So, basically, a FEES clinic is going to pay the same as a therapy‐led clinic, as a consultant‐led clinic. That … should make the management attitude more flexible’ [P11]
Time and workload	‘I've got a limited number of spaces. … We can't see every single person ourselves’ [P1]
Cost	‘You've probably heard of the Accelerator Programme and money that's been made available to Trusts, to try and deal with the backlog of patients’ [P8]
Efficiency	‘everyone had this general feeling that there were two groups of two week wait patients. The ones that we probably should be seeing and the ones that were a bit of a waste of time’ [P2]
Equipment and resources	‘ [existing advancements in] the development of videoscopes and being able to record’ [P1]
Workforce and sustainability	‘some places where you've got a strong speech and language department … where they would do that and they'd be involved’ [P2]
Service models	‘Started … in a parallel setting with [SLT] she interviewed and endoscoped the patients, made clinical decisions and then discussed the cases with myself … we reviewed the photographs, … videos, … the management decisions that were made’’ [P7]
Precedent	‘we'd been running SLT clinics in different forms for many years nonmedical requesting has become more acceptable’ [P1]
Need	‘a rising number of two week wait referrals, most of which probably don't need to see an ENT consultant’ [P1]

**TABLE 5:** Theme 3: Challenges.

**Sub‐theme**	**Examples**
Governance	‘you may be lucky and never come across a bad case, but when it happens, then you will very quickly find the protections are non‐existent’ [P11]
Training and supervision	‘legally, no consultant, however enthusiastic, is in a position to sign off a speech and language therapist to make a mucosal diagnosis’ [P11]
SLT competence	‘it might be harder for them to sort out the wheat from the chaff … patients who come along with globus tend to get over investigated by less experienced people’ [P4]
SLT interest	‘why have people gone into speech and language therapy? … because there's something about it that suits their personality, because they don't want to take that level of responsibility’ [P6]
Patient experience	‘Unless you're seeing me, the elderly, old male consultant, then you feel like you're not getting the best treatment’ [P10] ‘a lot of the time they don't think I'm the doctor … patients have a stereotype … you've got to be a man … if you're … a woman, are you a nurse? … my only concern … having more women in speech and language therapy … is how that perception and how that barrier is married up, so that patients feel confident when they come in that you have the expertise’ [P9]
MDT support	‘some of my colleagues still don't like the idea of using a community clinic, and everything needs to be seen in a consultant clinic’ [P1]
Organizational support	‘how do you take [stakeholders] along a journey when they've traditionally always had an entrenched view on what they define to be the roles and their place in society, or in their departments, or in their MDTs?’^a^ [P9]
Time and workload	‘I just don't think that [SLTs] have the time for it’ [P2]
Cost	‘it doesn't convert to theatre and it doesn't convert to any other earnings for us, it doesn't really help build us a case for saying, well, we need another speech therapist because it would give these benign guys a good service’ [P2]
Efficiency	‘The whole reason … is to speed up the diagnosis of cancers, and to some extent, [SLT] involvement is shifting the emphasis and moving away from that’ [P6]
Equipment and resources	‘where it will otherwise fail is I have my normal clinic and then I have a speech therapist now doing their clinic, all of us sharing one or two scopes. It's just not going to work. So, I will see less, they will see less. So, we need to have extra equipment’ [P1]
Workforce and sustainability	‘it's also not true that our therapy colleagues are sitting around twiddling their thumbs, and there is this massive resource that suddenly we are going to tap into. So, their staffing and the support that they need, and the numbers they need to take this on, need to be very carefully considered’ [P11]
Service models	‘every centre has their unique way of dealing with this and have all of the different specialities and allied health professionals … it will always be difficult to come up with a perfect, unified pathway for everywhere’ [P10]
Precedent	‘we've been using nurse practitioners in [place] for decades, and they're well established, but I don't actually think they're terribly efficient’ [P4]
Need	‘I'm not overrun with other work that I need to get done. So, I probably would have less of an impetus to do [SLT‐led clinics]’ [P1]

*Note*: ^a^Multidisciplinary teams (MDT).

**TABLE 6:** Theme 4: Requirements.

**Sub‐theme**	**Examples**
Governance	‘a local protocol. Some people are happy to not review it. Other people would … say they'd rather see everyone, see all the videos. I don't think there is a right or wrong answer’ [P1] ‘certainly when you had non‐medical people doing endoscopies, you should record them for their own protection’ [P4]
Training and supervision	‘[for] endoscopy, the number is 200. For colonoscopy, the number is 300. I would expect roughly the same numbers for mucosal diagnosis … you can do it whilst delivering the service at speed … you could basically get to your 200, 300 very quickly’ [P11] ‘we can standardise the video to say, *I want to see* …’ [P1] '[would need a] protocol or a clear pathway for ultrasound and MRI’ [P1] ‘[it would be] much easier for people who aren't very experienced to have a helping hand close by’ [P4]
SLT competence	‘once you feel your speech and language colleague is comfortable, and you feel they're competent, and you feel they're happy to potentially sit in a parallel clinic next to you, that is the clinic that could run’ [P9]
SLT interest	‘you should be asking the speech therapist whether they feel that picking up a lot of functional dysphonia would be an advantage’ [P4]
Patient experience	‘the vast majority of them just want … reassurance that there's nothing there. So, … we will let the patient know within 48 hours of the endoscopy what the result is’ [P2]
MDT support	‘to instil confidence to the wider allied health teams who may also have some doubt whether this is a model we should go with’ [P9]
Organizational support	‘[it needs] a national governance framework through HEE that says, pharyngolaryngeal endoscopy is … something that non‐medical clinicians are able to practice, and here is the training programme … and … the competencies’ [P11]
Time and workload	‘SLT [are] … obviously quite stretched already … it's a case of obviously creating a job plan’ [P3]
Cost	‘you'd have to check with the … local CCGs’^a^ [P4]
Efficiency	‘get the patient off the cancer pathway effectively, and also you get them into an intervention that's helpful’ [P4] ‘pre‐record and then have a consultant review. And that built into the consultant clinic time’ [P9]
Equipment and resources	‘I would have a nurse or an HCA^b^ cleaning my scope, the SLT has got to do it themselves. … It [is] … counterintuitive that you're now training somebody up and … them … cleaning the scope, rather than seeing other patient’ [P1]
Workforce and sustainability	‘Ideally … a dedicated, focussed core group of SLTs doing this day in, day out. … Otherwise, you're just effectively repeating what we have with the trainee group, this evolving and then changeover’ [P9]
Service models	‘I would see these as running more in the community’ [P1] ‘start thinking about what would you do with the ones that needed surgical treatment, like polyps or papillomas’ [P4]
Precedent	‘in clinic every week … face‐to‐face scope … over a six month period, probably 80 to 100’ [P3] ‘It took her 50 endoscopies … for her to become autonomously competent’ [P2] ‘suggest if it's protocolised … we've got a reflux protocol that our physician's associate and clinicians tend to you use here’ [P3]
Need	‘[a need to] show that … [SLTs in the role] was necessary. … And that it wouldn't disadvantage patients’ [P4]

*Note*:

^a^ Clinical commissioning groups (CCGs) are bodies that commission health services for each geographical region across the UK. More recently renamed integrated care boards since July 2022.

^b^ A healthcare assistant (HCA) is a team member who provides health and care support for patients and is not registered with the Nursing and Midwifery Council.

Many subthemes appeared within more than 1 theme. For example, an *equipment and resources positive* was that SLTs already possess the equipment to perform nasendoscopy (Table 3). Developments in the ability to record with this equipment would also *facilitate* success of an SLT‐led model (Table 4). However, operationally the number of scopes required per clinic for both ENTs and SLTs in parallel was felt to pose a *challenge* (Table 5), with a *requirement* for increased nursing support to sterilize the scopes between patients (Table 6) to fully realize the potential increased efficiency. This demonstrates the complexity of implementing new clinic models whereby the existence of equipment and skills may not translate into sufficient resources to implement them in clinical settings.

## DISCUSSION

Participants were supportive in principle of an SLT‐led model for low‐risk patients with suspected HNC. Some had already implemented an SLT‐led model, while others were considering doing so or had considered it previously. Some did not feel it suited their own service, but they were unopposed to the concept. Reasons for not utilizing SLTs included a lack of local workforce need; utilization of alternative health professionals in the extended role such as nurse or physician associate; desire for further governance; or a lack of SLT resource for example, number, perceived interest, or level of expertise/competency.

There was disparity among respondents regarding some aspects of the model and how it might be implemented at their own centres. Some ENT surgeons felt that an SLT‐led model would be ideal in a community setting to move care closer to home and increase uptake of services among patients who are reluctant to attend hospital based services; while others felt a community setting would present an issue for supervision and governance. Perceived patient experience also varied; for example, respondents already implementing an SLT‐led model reported positive patient experiences consistent with published data (Butler et al., 2023; Occomore‐Kent & Slade, 2021), while others not adopting the model felt that patients would wish to see a consultant for suspected cancer clinic appointments which has not been the case in the existing pilot studies above. Other factors such as age and gender were discussed, with one respondent indicating that patients believe that best care is provided by the ‘old male consultant’ specifically. Another respondent affirmed this with their own experience as a female ENT consultant suggesting that this may pose similar issues for a female dominated profession, such as SLT, adopting the proposed extended role. With regards to efficiency, some surgeons felt it acceptable and expected for SLTs to assess fewer patients per clinic to allow a more detailed assessment; perceiving this as beneficial to patients and their pathway overall. Others felt strongly that the clinic is solely for a binary cancer diagnosis and expressed unfairness for those patients referred through the routine pathway who must wait longer for such a detailed examination.

Another key difference related to governance requirements. While some ENT surgeons felt that the role and job description could be adjusted for the existing SLT skill‐set; others felt that anyone taking on the extended role must be competent in all existing aspects of it; for example, full neck examination and referral to radiology. Where SLTs cannot take on the full responsibilities of the clinic, it was felt to undermine the intended cost, time, and efficiency savings. Similarly, some ENT surgeons felt that reviewing every nasendoscopy recording was relatively efficient compared with conducting the entire consultation; while others felt this was unnecessary if an SLT was deemed competent. The extent of training, the competencies required, the method of evidencing competence, and the ongoing supervision requirements differed among respondents. Patient experience and pathway efficiency of the SLT‐led model require further exploration and evaluation; with the role description itself and its competencies needing further research and clarity. While some professional statutory regulatory body (PSRB) skills frameworks exist to support core aspects of patient assessment (Wallace et al., 2020; Jones et al., 2020), other extended skills that some respondents felt to be necessary are not currently included in any SLT‐specific skills frameworks or pre/post‐registration training for example, neck examinations.

With regards to the SLT workforce, some ENTs reported their SLT colleagues were enthusiastic about extending their role in this way affirming recent SLT workforce consultation (Bradley & Patterson, 2021; Ocomore‐Kent et al., 2021), while others perceived low interest among an already overworked SLT team. This may reflect SLTs’ opinions that a dedicated workforce and job description are required to undertake this role (Bradley & Patterson, 2021; Occomore‐Kent et al., 2021), rather than a lack of enthusiasm for the concept. Some ENTs working alongside highly experienced SLT colleagues felt their SLTs already have many of the prerequisite skills needed to perform this role and, in some cases, SLTs already do so at their centres successfully. These skills included laryngeal examination and flexible nasendoscopy. Other respondents felt their SLT colleagues locally did not possess these skills and would require significant upskilling, sometimes in the context of low enthusiasm for the role.

### Strengths and limitations

While the diversity of our sample was considered and maximized during recruitment, as well as agreement that no new themes were occurring after 11 interviews, the number of participants is relatively small and the findings may not represent all viewpoints of consultant ENTs. The geographical diversity of the sample is a strength of this study. Participants were not presented with their transcripts or quotations for checking, however verbatim quotes were used in the reporting to minimize subjective interpretation. Analysis was conducted through discussion among a diverse team of professionals on the research team from de‐identified full transcripts to minimize bias. The interviews were conducted by an ENT surgeon and a GP to promote honest and open sharing of views about SLTs in this role.

## CONCLUSIONS

No single clinical model that would fit all centres’ needs was identified but the following general principles for low‐risk SLT‐led suspected HNC referral clinics may be concluded from these interviews:

- The role should be performed by SLTs with experience working in the ENT and HNC fields, with competence in laryngeal examination and performing flexible nasendoscopy.
- The role should be incorporated into job plans of dedicated staff and not absorbed by the general workload of the wider SLT department.
- An outline of the duties for SLTs leading low‐risk suspected HNC clinics, that are beyond the current ENT/HNC SLT role, should be clarified.
- A training programme, competency framework and supervision structure should be created, similar to that created by other national Allied Health Practitioner training initiatives, for example, endoscopy.
- Professional and regulatory body recognition of the role is necessary to protect SLTs, ENTs and patients, and an appropriate job description and remuneration should be identified to reflect the responsibility and skill required by the role.
- Further research is needed to evaluate outcomes of implementing SLT‐led clinics, such as efficiency, care pathway changes, patient experience, safety, clinical outcomes and ENT/SLT acceptability.

## CONFLICTS OF INTEREST STATEMENT

The authors declare no conflicts of interest.
